# Reducing Metal Artifacts in Clinical Photon Counting Detector Computed Tomography—A Phantom Study of an Exemplary Total Hip Arthroplasty

**DOI:** 10.1007/s00256-024-04820-2

**Published:** 2024-11-19

**Authors:** Roy P. Marcus, Georg C. Feuerriegel, Adrian A. Marth, Sophia S. Goller, Daniel Nanz, Julian Anhaus, Reto Sutter

**Affiliations:** 1 Department of Radiology, Balgrist University Hospital, Zurich, Switzerland; 2 Faculty of Medicine, University of Zurich, Zurich, Switzerland; 3 Swiss Center for Musculoskeletal Imaging, Balgrist Campus, Zurich, Switzerland; 4 Siemens Healthineers, Forchheim, Germany

**Keywords:** Photon-counting computed tomography, Metal artifact, Tin filter, Ultra-high-resolution, Virtual monoenergetic imaging, Total hip prosthesis

## Abstract

**Objective:**

To examine how different photon-counting detector (PCD) CT scanning and reconstruction methods affect the volume of metal artifacts and image quality for a hip prosthesis phantom.

**Methods:**

A titanium and cobalt-chromium-molybdenum-alloy total hip prosthesis phantom was scanned using a clinical PCD-CT with a constant tube potential (140 kV) and Computed-Tomography-Dose- Index (7 mGy). Different scan settings were used: with/without tin-filter (Sn), with/without ultra-high resolution (UHR), both individually and combined, resulting in four modes: Quantumplus (Standard), UHR Quantumplus (HighRes), QuantumSn (Standard-Tin) and UHR QuantumSn (HighRes-Tin). Reconstructions included virtual monoenergetic images (VMI) spanning 40–190 keV and polychromatic images, with/without iterative metal artifact reduction (MAR). Artifact volumes rendered in a 3D-printing software were quantified in milliliters (ml), and image quality was evaluated using a Likert score.

**Results:**

Polychromatic reconstruction: Tin-filter reduced artifact volumes (298 (Standard-Tin) vs. 347 ml (Standard) and 310 (HighRes-Tin) vs. 360 ml (HighRes)). The smallest artifact volume was measured in HighRes MAR (150 ml).

VMI reconstruction: The smallest artifact volume was measured in Standard 130 keV (150 ml) and HighRes 130 keV (164 ml) and in Standard-Tin 120 keV (169 ml) and HighRes-Tin 120 keV (172 ml). MAR further reduced the artifact volumes to 130 ml (Standard 150 keV MAR) and 140 ml (HighRes 160 keV MAR).

Image quality was rated best for Standard 65 keV MAR, polychromatic HighRes MAR, Standard 100 keV MAR, polychromatic Standard-tin MAR, HighRes-tin 100 keV and polychromatic HighRes-tin.

**Conclusion:**

Combining tin-filter, UHR and MAR in VMI or polychromatic images achieve the strongest artifact reduction.

**Supplementary Information:**

The online version contains supplementary material available at 10.1007/s00256-024-04820-2.

## Introduction

Photon-counting-detector computed tomography (PCD CT) has transformed clinical cross-sectional imaging [1–3]. Unlike EID CT, which registers signal strengths in proportion to the total photon energy delivered to a detector element, each element of the novel PCD counts the incoming photons and sorts the corresponding events into predefined predesignated bins according to the energy of the incoming photons [4]. Incoming photons with an energy below the lowest threshold can be ignored, which effectively eliminates electronic noise and results in images with a superior signal-to-noise-ratio [5–7]. Compared to energy integrating detector CT (EID-CT), spatially resolved multi-energy information enables spectral imaging and post-processing in PCD CT without requiring an additional scan or parallel acquisition at a different voltage potential [4, 8–10].

Effective metal artifact reduction is crucial for optimizing clinical image quality, particularly in musculoskeletal radiology. By minimizing metal- induced distortions, this technique significantly improves the visualization of critical anatomical structures, such as bone-metal interface, and enhances the detection of hardware loosening and periprosthetic fractures near the implants [11, 12]. To date, a limited number of studies have evaluated the technical effectiveness of metal artifact reduction techniques in PCD CT [13–18], primarily focusing on virtual monoenergetic and iterative metal artifact reduction (MAR) image reconstruction techniques [17–19]. To date, no study has systematically evaluated the potential of tin prefiltration and/or ultra-high-resolution (UHR) acquisitions available on clinical PCD CT. Specifically, the impact of an increased photon count on the UHR detector (based on an element area of 0.25 × 0.25 mm) on the reduction of metal artifacts has not been examined.

The purpose of this study was to quantitatively assess the impact of clinically available combinations of scanning modes and image-reconstruction methods on objectively measured metal artifact volumes in PCD CT images of a hip prosthesis phantom.

## Materials And Methods

### Phantom

**Fig. 1:**
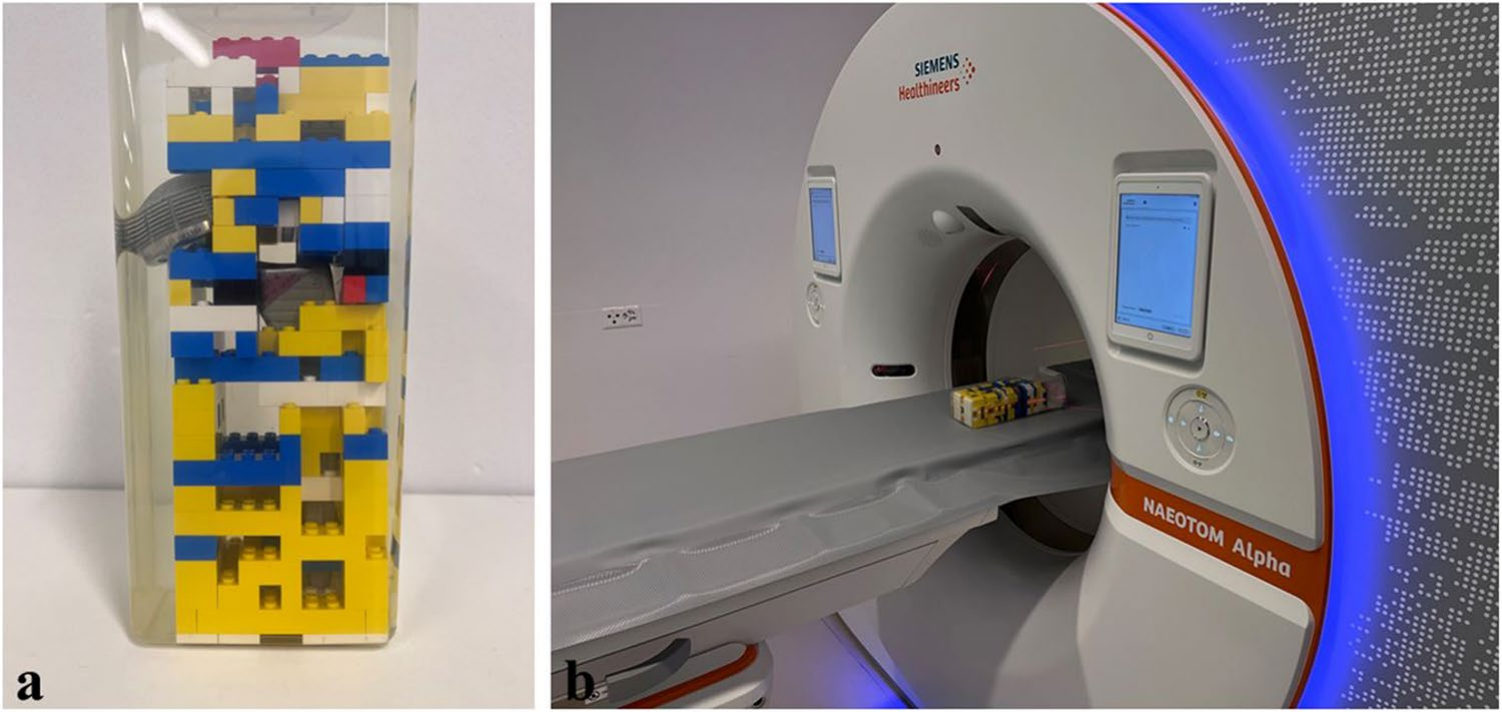
**a** Total hip prosthesis encased in plastic brick scaffold and placed in a plastic container filled with distilled water. **b** The phantom on the table of the 1st generation clinical photon-counting detector computed tomography (PCD CT) scanner

A total hip prosthesis (Titanium Quadra-H stem, titanium Versafit-CC cup, femoral head replacement with cobalt-28 chromium-6 molybdenum alloy casting; Medacta, Castel San Pietro, Switzerland) with a polyethylene inlay was encased in a plastic brick system (The LEGO® Group, Billund, Denmark) and placed in a plastic container filled with distilled water (Fig. 1).

### Image Acquisition

All images were acquired with a clinical PCD CT scanner (Naeotom Alpha, Siemens Healthineers, Forchheim, Germany) using four distinct acquisition modes.

(I) Quantumplus (Standard): Acquisition with an in-plane resolution of 0.2 mm, encompassing the full spectral range from 20 to 190 keV.
(II) Ultra-High resolution Quantumplus (HighRes): Ultra high resolution (UHR) mode with an in-plane resolution of 0.11 mm, encompassing the same full spectral range as described in mode (I) above. This mode allows for increased sharpness and detailed depictions [6, 20, 21].
(III) QuantumSn (Standard-Tin): Acquisition with an in-plane resolution of 0.25 mm and the use of a tin filter with a thickness of 0.4 mm, which eliminates low energy photons, resulting in an acquisition with a reduced spectral range from 40 to 190 keV.
(IV) Ultra-High resolution QuantumSn (HighRes-Tin): UHR mode with an in-plane resolution of 0.11 mm, acquired with tin filter as in mode (III), resulting in an acquisition with a reduced spectral range.

For all acquisition modes, the tube potential was set to 140 kV_p_ with a CTDI_vol_ dose index of 7 mGy, pitch of 0.8, and rotation time of 0.5 rotation / second. The CTDI_vol_-value corresponded to the value obtained in the majority of examinations performed on our clinical EID-CT. Collimation of 120 × 0.2 mm was set for UHR acquisitions and 144 × 0.2 mm for non-UHR acquisitions, respectively.

### Image Reconstruction

The following image series were reconstructed with a slice thickness of 2 mm and an increment of 1 mm, a soft body (B40) kernel, an iterative strength of 3 (out of 4) (Quantitative Iterative Reconstruction, Siemens Healthineers, Forchheim, Germany), a 512 matrix and a 100 mm field of view (FOV):

- Polychromatic: Low-energy-threshold polychromatic images encompassing photon energies of 20–120 keV.
- Virtual monoenergetic images (VMI): reconstructed for specific energy levels [22].
- from 40 to 190 keV with an increment of 10 keV for acquisition modes (I) and (II) and
- from 60 to 190 keV with increments of 10 keV for acquisition modes (III) and (IV).

In addition, the factory preset monoenergetic images for soft-tissue imaging were reconstructed at 65 keV for mode (I) and 85 keV for mode (III).

All raw data series were reconstructed using a commercially available iterative metal artifact reduction program (MAR,, Siemens Healthineers, Forchheim, Germany).

### Quantitative Evaluation

**Fig. 2:**
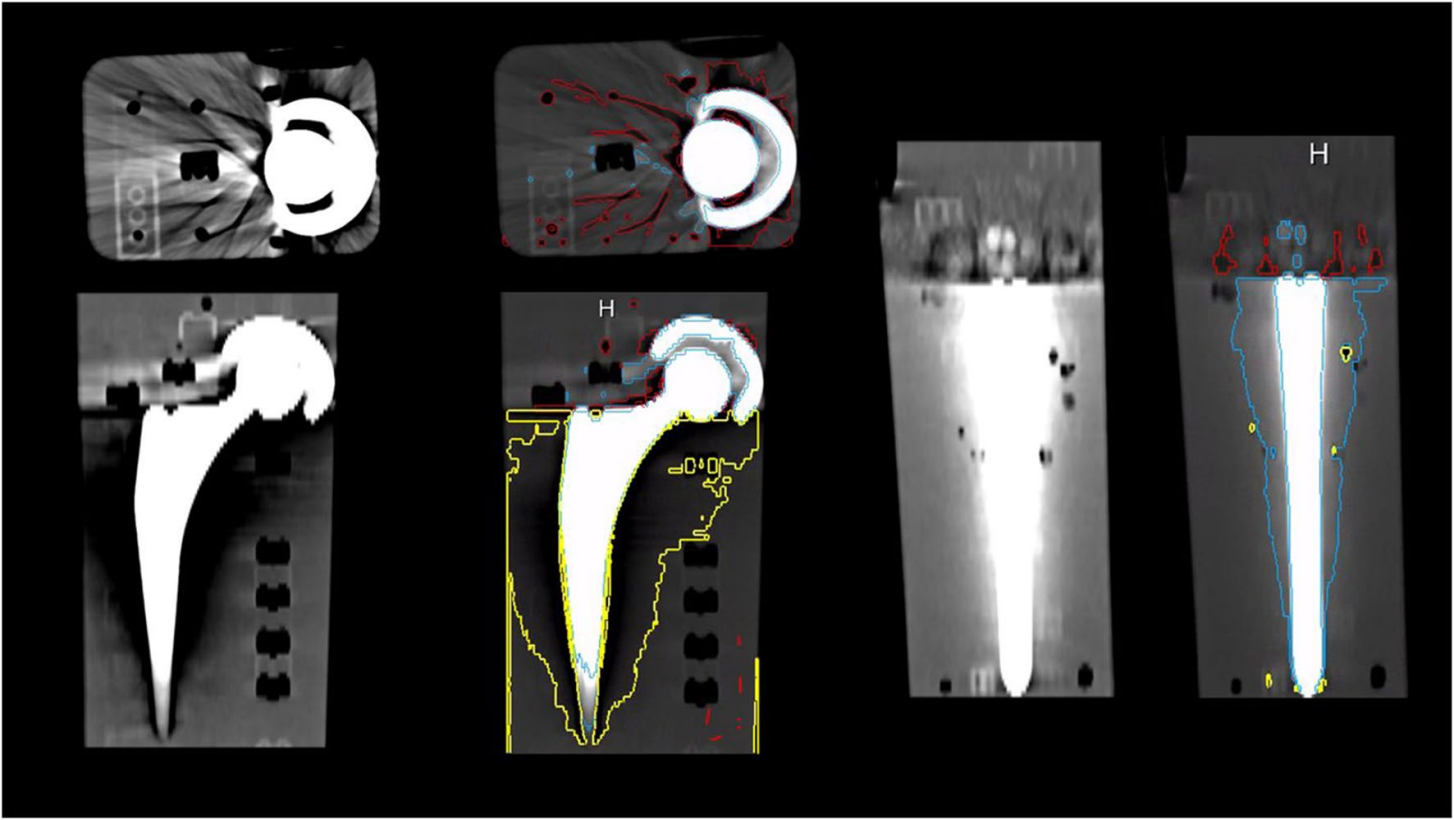
Exemplary PCD CT images of the total hip prosthesis phantom were acquired at 140 kV with a tin filter in HighRes mode and reconstructed at 85 keV (factory preset reconstruction) using a soft body B40 kernel. Volumetric segmentation of the differently attenuated regions with metal artifacts is shown in color: yellow = hypo-attenuation artifact around the stem, red = hypo-attenuation artifact around the cup and head, and blue = hyper-attenuation artifact around the cup, head, and stem. PCD CT = photon-counting detector computed tomography. UHR = ultra-high resolution

All image series were loaded into a 3D printing software (MM 3D Printing version 1.2.7, Siemens Healthineers, Erlangen, Germany) implemented in a clinical post-processing environment (syngo.via VB 60, Siemens Healthineers, Erlangen, Germany). A board-certified and fellowship-trained radiologist with 10 years of clinical experience (R.P.M.) semi-automatically segmented the hyper- and hypo-attenuated artifact volumes as described in [23]. The plastic bricks exhibited an attenuation range of -5 to + 5 Hounsfield units (HU), demonstrating comparable properties to distilled water, thereby exerting no discernible impact on the metal artifact volumes. Thresholds for hyper-attenuated artifacts were set between 165 and 1300 (HU), whereas those for hypo-attenuated artifacts were set between -750 and -25 HU. This allowed the software to automatically segment the hyper- and hypo-attenuated artifact volumes. Volumes that were falsely included or missed by simple thresholding were manually deleted or added. The software automatically computed image volumes containing hyper- and hypo-attenuated artifacts, and the total artifact volume was determined as the combined sum of these volumes (Fig. 2).

### Qualitative Evaluation

Qualitative image evaluation was conducted on a clinical PACS viewer (Merlin Diagnostic Workcenter v.7.1.222429, Phönix-PACS, Freiburg, Germany) independently by two board-certified radiologists with musculoskeletal fellowship training and with 10 and 6 years of clinical experience (R.P.M and S.S.G), and by two subspecialty fellows in musculoskeletal radiology, each with 5 years of clinical experience (A.A.M. And G.C.F.). All readers were blinded to the acquisition and reconstruction settings.

During the first stage, each image was evaluated for the degree of artifacts, prosthesis delineation, and presence of a synthetic/aquarelle-like image impression. The severity of artifacts was graded using a 5-point Likert score, similar to the scale introduced by Mohammadinejad et al. [24] 1 = no streak artifacts, 2 = minimal streak artifacts with no effect on surrounding structures, 3 = minimal streak artifacts that affect the surrounding structures, 4 = moderate streak artifacts that considerably affect the surrounding structures, and 5 = severe streak artifacts that significantly affect the surrounding structures (Supplementary Fig. 1). Assessment of prosthesis delineation also employed a 5-point Likert score: 1 = prosthesis is perfectly delineated, 2 = minimal impairment that does not affect prosthesis delineation, 3 = minimal impairment that affects prosthesis delineation, 4 = moderate impairment that considerably affects prosthesis delineation, and 5 = severe impairment that significantly affects prosthesis delineation (Supplementary Fig. 2). For the assessment of synthetic/aquarelle-like image impression, the following 5-point Likert score was employed: 1 = no synthetic/aquarelle-like image impression; 2 = minimal synthetic/aquarelle-like image impression, which does not affect the image quality; 3 = minimal synthetic/aquarelle-like image impression, which does affect the image quality; 4 = moderate synthetic/aquarelle-like image impression, which does considerably affect the image quality; and 5 = severe synthetic/aquarelle-like image impression, which does significantly affect the image quality (Supplementary Fig. 3).

The second stage was performed after a 30-day interval to mitigate potential recall bias, in which each image series under examination was placed next to a reference image series acquired in Q + (mode I) and reconstructed using VMI at 65 keV (factory preset reconstruction setting) without iMAR. All four readers had to determine in consensus whether they preferred the examined or reference images.

### Statistical Analysis

Quantitative values were measured in milliliters [ml] with differences shown as percentages. Qualitative scores are displayed individually and calculated as the mean. Fleiss kappa (κ) coefficient was calculated to assess the inter-rater reliability [25]. Agreement values were interpreted as follows: κ = 0 – 0.2 (none), κ = 0.21 – 0.39 (minimal), κ = 0.4 – 0.59 (weak), κ = 0.6 – 0.79 (moderate), κ = 0.8 – 0.9 (strong) and κ > 0.9 (almost perfect) [26]. The significance level was set at P < 0.05. Statistical analysis was conducted using IBM SPSS Statistics for Windows, Version 29.0. (IBM Corp, Armonk, NY.) and the graphs were generated accordingly.

## Results

In total, 132 reconstructions were evaluated, as summarized in Supplementary Table 1.

Acquisitions are documented as follows: Polychromatic images are denoted by the acquisition modes: Standard, Standard-Tin, HighRes or HjghRes-Tin. Virtual monoenergetic images are labelled with the acquisition mode followed by the monoenergetic level (e.g. Standard-Tin 90 keV). The application of MAR is indicated as a suffix for both polychromatic and virtual monoenergetic images (e.g. Standard-Tin MAR for polychromatic Standard-Tin acquisition with MAR or Standard-Tin 90 keV MAR for a Standard-Tin acquisition reconstructed at a monoenergetic level of 90 keV with MAR).

### Quantitative Evaluation

#### Polychromatic CT Reconstruction

The use of tin filter reduced the total artifact volume by 14% for both HighRes- and Standard images. The total artifact volume mainly consisted of a hypo-attenuating component, ranging from 73.5 to 80%.

**Fig. 3:**
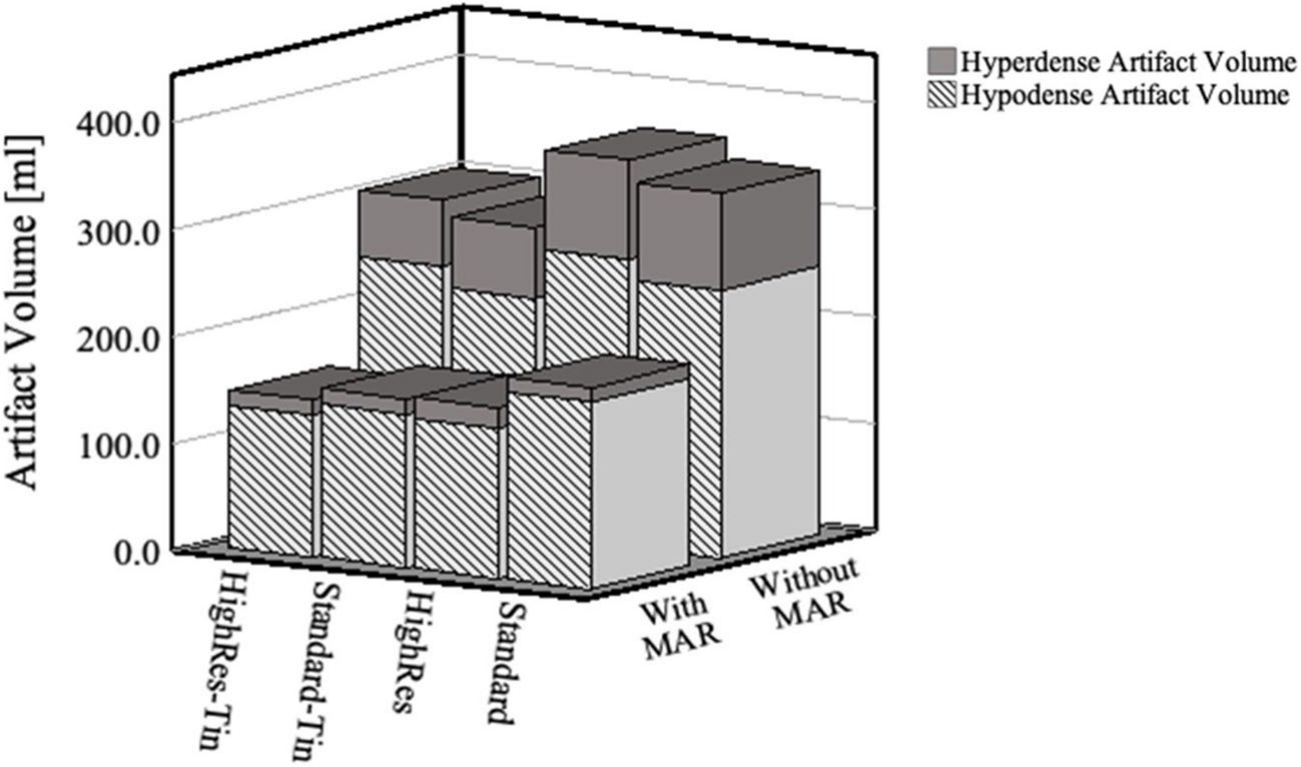
Bar graph illustrating hypo- and hyper-attenuation artifact volumes for each of the four acquisition modes at PCD CT reconstructed in the polychromatic (T3D) mode, both with and without MAR. The use of a tin filter (HighRes-Tin and Standard-Tin) resulted in smaller total artifact volumes. Use of the UHR option resulted in larger artifact volumes. MAR reduced all metal artifact volumes, with the largest effect on the hyper-attenuated component. Standard = Quantumplus; Standard-Tin = QuantumSn; HighRes = UHR Quantumplus; HighRes-Tin = UHR QuantumSn. PCD CT = photon-counting detector computed tomography; Sn = tin filter. UHR = ultra-high resolution

The smallest total artifact volume was measured for Standard-Tin (298 ml). HighRes-Tin showed a slightly larger artifact volume, when compared to Standard-Tin (310 ml), followed by Standard (347 ml), and HighRes (360 ml), (Tables 1 and 2 and Fig. 3).

**Table 1:** Hip prosthesis-induced artifact volume in Standard and HighRes acquisitions with and without MAR. Volumes are in ml. Standard = Quantumplus; HighRes = UHR Q + ; MAR, iterative metal artifact reduction; UHR, ultra-high resolution; kV, kilovolt

keV	Protocol	Total artifact volume		Hypo-attenuated artifact volume		Hyper-attenuated artifact volume		Protocol	Total artifact volume		Hypo-attenuated artifact volume		Hyper-attenuated artifact volume	
	**Standard**	Without MAR	With MAR	Without MAR	With MAR	Without MAR	With MAR	**HighRes**	Without MAR	With MAR	Without MAR	With MAR	Without MAR	With MAR
40		468	383	253	363	215	20		472	445	273	429	198	15
50		416	285	270	270	146	15		410	329	273	316	138	12
60		353	229	249	215	104	14		373	247	269	233	104	13
65		336	188	246	175	89	13							
70		308	187	231	171	77	16		323	215	247	200	77	15
80		258	171	204	154	53	17		264	197	211	181	53	16
90		216	155	184	140	32	15		224	173	194	159	30	14
100		183	148	167	136	16	12		190	165	174	153	16	12
110		166	139	154	127	12	11		184	159	172	148	12	11
120		160	138	149	127	10	11		169	152	159	141	10	11
130		150	134	140	123	10	11		164	148	155	137	10	11
140		153	134	143	123	10	11		164	146	154	135	10	11
150		161	130	150	119	11	12		162	144	151	133	11	12
160		152	136	141	124	11	12		162	140	152	128	11	12
170		158	130	146	118	12	12		165	141	153	130	12	12
180		161	134	148	122	12	12		168	143	156	131	12	12
190		167	130	154	118	13	12		166	142	153	130	13	12
Polychromatic		347	169	255	152	92	16		360	156	267	138	92	19

**Table 2:** Hip prosthesis-induced artifact volumes in Standard-Tin and HighRes-Tin acquisitions with and without MAR. Volumes are in ml. Standard-Tin = QuantumSn; HighRes-Tin = UHR QuantumSn; Sn = tin-filter; MAR = iterative metal artifact reduction; UHR = ultra-high resolution; kV = kilovolt

keV	Protocol	Total artifact volume		Hypo-attenuated artifact volume		Hyper-attenuated artifact volume		Protocol	Total artifact volume		Hypo-attenuated artifact volume		Hyper-attenuated artifact volume	
	**Standard-Tin**	Without MAR	With MAR	Without MAR	With MAR	Without MAR	With MAR	**HighRes-Tin**	Without MAR	With MAR	Without MAR	With MAR	Without MAR	With MAR
60		528	242	394	226	134	16		583	324	464	313	119	12
70		441	180	332	164	109	16		453	244	356	230	97	13
80		347	166	266	150	81	16		344	199	270	185	74	14
85		279	156	212	141	66	15							
90		259	155	206	140	53	13		260	179	215	165	46	14
100		190	143	167	130	23	12		199	166	180	154	19	13
110		169	142	156	130	13	12		183	163	171	151	12	12
120		169	142	158	130	11	11		172	156	162	145	10	12
130		179	137	169	126	10	11		177	153	168	142	9	11
140		187	137	178	126	10	11		183	147	174	136	9	11
150		189	134	179	123	10	11		187	148	178	137	9	11
160		191	136	181	126	10	11		183	146	174	135	9	11
170		200	137	189	126	11	11		189	143	179	131	10	11
180		200	134	188	123	12	11		188	142	178	130	10	11
190		203	134	190	123	13	11		191	140	180	129	11	11
Polychromatic		298	160	232	145	66	14		310	150	248	135	63	15

The largest total metal artifact reduction with MAR was achieved for HighRes (total artifact volume of 160 ml with MAR vs. 360 ml without MAR), followed by HighRes-Tin (total artifact volume of 150 ml with MAR vs. 310 ml without MAR), Standard (total artifact volume of 169 ml with MAR vs. 347 ml without MAR), and Standard-Tin (total artifact volume of 160 ml with MAR vs. 298 ml without MAR). MAR reduced the hyper-attenuated component by 76.2% to 82.6% and the hypo-attenuated component by 37.5% to 48.3%, respectively.

#### Virtual Monoenergetic Imaging Reconstructions

**Fig. 4:**
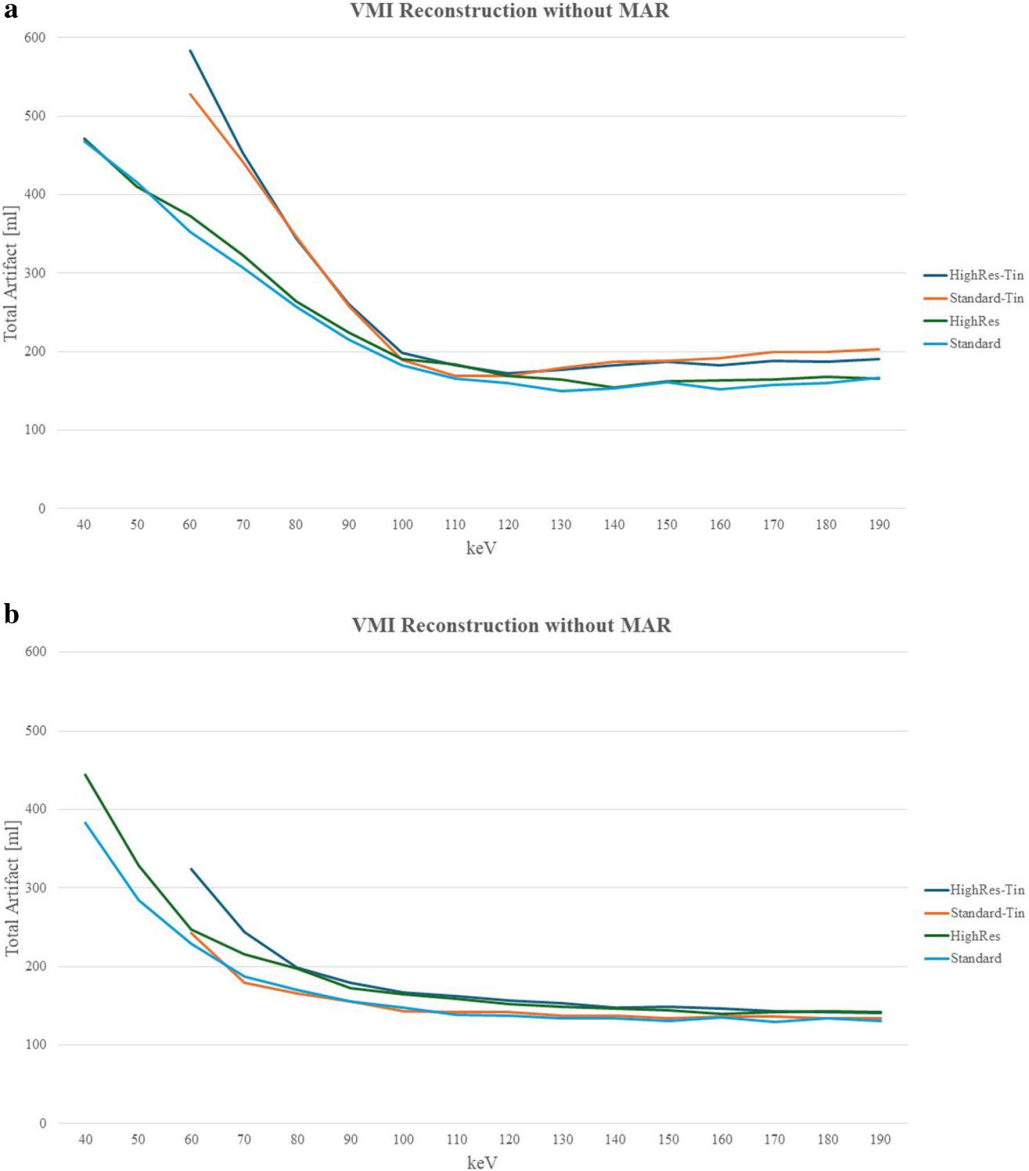
Total artifact volume at PCD CT in the VMI-reconstructed image series as a function of energy level, both without (**a**) and with iterative metal artifact reduction (MAR) (**b**). Notably, the introduction of a tin filter resulted in a marginal increase in total artifact volumes compared to acquisitions without tin filter. Furthermore, acquisitions conducted in the ultra-high-resolution (UHR) mode exhibited higher artifact volumes when compared with their non-UHR mode counterparts. The most substantial artifact volume was observed at the lowest attainable monoenergy level, whereas the smallest artifact volume was quantified within the energy range of 110–130 keV. The application of MAR successfully mitigated all artifacts; however, it also shifted the monoenergy level for the smallest artifact volume to a higher value. Standard = Quantumplus; Standard-Tin = QuantumSn; HighRes = UHR Q + ; HighRes-Tin = UHR QuantumSn; PCD CT = photon-counting detector computed tomography, Sn = tin filter

Using the tin filter resulted in a 49.6% increase of total artifact volume for Standard acquisitions and a 65.2% increase for HighRes acquisitions. As monoenergetic levels increased, the total artifact volume declined sharply across all acquisition modes, reaching a minimum at mononenergetic levels of 110–130 keV: Standard 130 keV (150 ml), HighRes 130 keV (164 ml), Standard-Tin 110 keV and Standard-Tin 120 keV (169 ml) and HighRes 120 keV (172 ml) (Tables 3 and 4, Fig. 4a).

**Table 3:** Visual assessment of artifacts, prosthesis delineation, and synthetic image impression in Standard and HighRes acquisitions with and without MAR evaluated by four readers. Mean values are depicted. The assessment was performed using a 5-point Likert score, where a score of 1 denotes no streak artifacts in artifacts, perfectly delineated prosthesis in the prosthesis delineation category, and no synthetic/aquarelle-like image impression in the synthetic image impression category. A score of 5 signified the presence of severe streak artifacts that significantly affected the image quality in the artifact category, severe impairment that significantly affected the prosthesis delineation in the prosthesis delineation category, and severe synthetic/aquarelle-like image impression that significantly affected the image quality in synthetic image quality. The preference over a reference Standard 65 keV image series was also assessed. Standard = Quantumplus; HighRes = UHR Quantumplus; MAR, iterative metal artifact reduction; UHR, ultra-high resolution; kV, kilovolt

Protocol	keV	Artifacts		Prosthesis delineation		Synthetic image impression		Preference?	
		Without MAR	With MAR	Without MAR	With MAR	Without MAR	With MAR	Without MAR	With MAR
Standard	40	4	3.75	5	4.25	5	5	No	No
	50	4.5	3.5	5	4	2.75	3.25	Yes	Yes
	60	5	3.25	5	3	2	3	No	No
	65	4.25	2	4.5	2	1	1.5	No	Yes
	70	4	3	5	3	2	2	No	Yes
	80	4	3	4.75	3	2	2	No	Yes
	90	4	3	4.25	3.5	2.25	2	No	Yes
	100	4.25	3.25	4.5	4	4	3.25	No	No
	110	4.75	3	5	4	4	4	No	No
	120	4.75	3	5	4	5	4	No	No
	130	5	3	5	4	5	4	No	No
	140	5	3.25	5	4	5	4	No	No
	150	5	3.25	5	4	5	4	No	No
	160	5	3	5	4.5	5	4	No	No
	170	5	3.25	5	4.75	5	4.75	No	No
	180	5	3.5	5	5	5	5	No	No
	190	5	3.5	5	5	5	5	No	No
	Polychromatic	4.25	2	4	4	1	1	No	Yes
HighRes	40	5	3.25	5	3.75	5	4	No	No
	50	5	3.25	5	4	5	4	No	No
	60	4.75	3	4.5	4	3	4	No	No
	70	4.25	3	4.25	3.25	2	3.5	No	Yes
	80	4	2.25	4	2	2	3	Yes	Yes
	90	4	2	4	2	3.25	3	No	No
	100	4.75	3	4	3.5	4	3	No	No
	110	4.5	3	4.25	3.75	4	3.75	No	No
	120	5	3	5	4	5	4	No	No
	130	5	3.25	5	4	5	4	No	No
	140	5	3.25	5	4.25	5	5	No	No
	150	5	3.75	5	5	5	5	No	No
	160	5	4	5	5	5	5	No	No
	170	5	4	5	5	5	5	No	No
	180	5	4	5	5	5	5	No	No
	190	5	4	5	5	5	5	No	No
	Polychromatic	4.25	2	4	2	1.5	1.75	No	Yes

**Table 4:** Visual assessment of artifacts, prosthesis delineation, and synthetic image impressions in Q-Sn and UHR Q-Sn acquisitions with and without iMAR evaluated by four readers. Mean values are depicted. The assessment was performed using a 5-point Likert scale, where a score of 1 denotes no streak artifacts in artifacts, perfectly delineated prosthesis in the prosthesis delineation category, and no synthetic/aquarelle-like image impression in the synthetic image impression category. A score of 5 signified the presence of severe streak artifacts that significantly affected the image quality in the artifact category, severe impairment that significantly affected the prosthesis delineation in the prosthesis delineation category, and severe synthetic/aquarelle-like image impression that significantly affected the image quality in synthetic image quality. The preference over a reference Standard 65 keV image series was also assessed. Results of the visual assessment of artifacts, prosthesis delineation, and synthetic image impressions of Standard-Tin and HighRes-Tin with and without MAR Standard-Tin = QuantumSn; HighRes-Tin = UHR QuantumSn; Sn, tin-filter; MAR, iterative metal artifact reduction; UHR, ultra-high resolution; kV, kilovolt

Protocol	keV	Artifacts		Prosthesis delineation		Synthetic image impression		Preference?	
		Without MAR	With MAR	Without MAR	With MAR	Without MAR	With MAR	Without MAR	With MAR
Standard-Tin	60	3.75	3	3.75	2	2	4	No	Yes
	70	5	4	4.5	2	2.75	3	Yes	Yes
	80	5	3	4.25	2	2.5	3	Yes	Yes
	85	4.25	3.25	4	2	2	2	Yes	Yes
	90	4	3	4	2	2	2	Yes	Yes
	100	3.25	2.25	3.5	2	2	2	Yes	Yes
	110	3	2.75	3.25	2.75	3.25	3	Yes	Yes
	120	3	3	3.75	3	3.75	3	No	No
	130	4.25	3	4	3.5	4	3.75	No	No
	140	4.75	3	4.75	4	4.75	4	No	No
	150	5	3	5	4	5	4	No	No
	160	4.75	3	5	4	5	4	No	No
	170	5	3.25	5	4	5	4	No	No
	180	5	3.5	5	4	5	4	No	No
	190	5	3.75	5	4	5	4	No	No
	Polychromatic	4	2	4.25	2	1	1.75	Yes	Yes
HighRes-Tin	60	5	4.25	4.5	4.25	2	3.25	No	No
	70	5	4	5	4	2	3	No	No
	80	4	3	4	3.5	2	3	No	Yes
	90	4	2.25	4	2	2	2	Yes	Yes
	100	3.5	2	3.5	2	2	2	Yes	Yes
	110	3	2.75	3.5	3	2.5	2.75	No	Yes
	120	3	3	3.75	3	4	3	No	Yes
	130	3	3	4	3.25	4	3	No	No
	140	3.75	3	4	3.5	4	4	No	No
	150	4	3.25	4	4	4	4	No	No
	160	4.5	3.25	4.75	4	4.75	4	No	No
	170	5	3.75	5	4	5	4	No	No
	180	5	4	5	4	5	4	No	No
	190	5	4	5	4	5	4	No	No
	Polychromatic	4.25	2	4	2	1	2	Yes	Yes

Compared to VMI without MAR reconstructions, the reduction in total artifact volumes with MAR reconstructions occurred more gradual, resulting in a flatter curve. The smallest total artifact volumes at higher monoenergetic levels were observed as follows: HighRes 160 keV (140 ml), Standard 150 keV (130 ml), Standard-Tin 150 keV (134 ml), and HighRes-Tin 190 keV (140 ml) (Tables 3 and 4, Fig. 4b).

MAR had the greatest impact on the hyper-attenuating component, reducing it by up to 87.8% in Standard-Tin and 92.4% in HighRes. The use of MAR increased the hypo-attenuated volume by 43.3% in Standard and by 57.1% in HighRes. However, MAR reduced the hypo-attenuated component by up to 32.7% in HighRes-Tin and by 42.7% in Standard-Tin.

### Qualitative Visual Evaluation

Tables 3 and 4 summarize the mean scores of the qualitative visual evaluation. The individual scores are summarized in Supplementary Table 2–5.

#### Artifacts

The exemplary images for each Likert score are shown in Supplementary Fig. 1. A score of 1 was not assigned to any of the reconstructions. VMIs between 65 and 100 keV and all polychromatic images reconstructed with MAR had minimal artifacts (mean scores of 2 – 2.25): Standard 65 keV MAR (mean score of 2), HighRes 80 keV (mean score of 2.25) and HighRes 90 keV (mean score of 2), Standard-Tin 100 keV (mean score 2.25), HighRes-Tin 90 keV (mean score of 2.25), and HighRes-Tin 100 keV (mean score of 2). Minimal artifacts (mean scores of 3–3.5) in images without MAR reconstruction were observed in HighRes-Tin 100 keV (mean score of 3.5), HighRes-Tin 110 keV (mean score of 3), HighRes-Tin 120 keV (mean score of 3), and HighRes- Tin 130 keV (mean score of 3), as well as for Standard-Tin 100 keV (mean score of 3.25), Standard-Tin 110 keV (mean score of 3), and Standard-Tin 120 keV (mean score of 3). All the VMI energy levels of Standard acquisitions with MAR were associated with a mean score of 3 – 3.75. Polychromatic reconstructions without MAR were associated with moderate artifacts (mean scores of 4 – 4.25). Severe artifacts (mean score of 5) were observed only in images that were not processed using MAR). The inter-rater reliability was moderate across all readers (κ = 0.723).

#### Prosthesis Delineation

The exemplary images for each Likert score are shown in Supplementary Fig. 2. All the reconstructions exhibited imperfect prosthesis delineations. HighRes MAR, Standard-Tin MAR, and HighRes-Tin MAR showed minimal artifacts in polychromatic images along the prosthesis border (mean score, 2). For the VMIs, a mean score of 2 was assigned to HighRes 80–90 keV MAR, Standard 60–100 keV MAR, HighRes-Tin 90–100 keV MAR and Standard 65 keV MAR. Minimal artifacts affecting the prosthesis border (score 3) included only VMIs. Except for Standard-Tin 100 keV (mean score of 3.5) and Standard-Tin 110 keV (mean scores of 3.25), the remaining VMIs with this score were all processed with MAR. The remaining polychromatic reconstructions, with and without MAR, showed moderate impairment of the prosthesis border. Severe degradation of the prosthesis representation included lower and higher VMI monoenergy spectra. The inter-rater reliability across all readers was moderate (κ = 0.775).

#### Synthetic / Aquarelle-like Image Impression

The exemplary images for each Likert score are shown in Supplementary Fig. 3. Standard 65 keV, and all polychromatic images without MAR reconstructions showed no synthetic image impressions (mean score of 1 – 1.5). Polychromatic images reconstructed with MAR displayed minimal synthetic image impression while maintaining good image quality (mean scores of 1.75—2). Most VMIs with a score of 2 were not reconstructed using MAR. Mean VMI rating for the factory preset Standard 65 keV MAR was 1.5. VMI ratings for Standard with and without MAR were all scored at 2 for a factory preset VMI setting of 85 keV. The remaining VMIs varied from 50 to 100 keV. Minimal synthetic image impression affecting image quality (score 3) was observed more often in VMIs reconstructed with MAR than in those reconstructed without MAR, especially at monoenergy levels ranging from 50 to 130 keV. Moderate synthetic image impression (score 3.75—4.5) was observed in most images reconstructed with MAR, while severe synthetic image impression (mean scores of 4.75—5) was observed in the majority of images not reconstructed with MAR. The inter-rater reliability was strong across all readers (κ = 0.873).

#### Image Preference

**Fig. 5:**
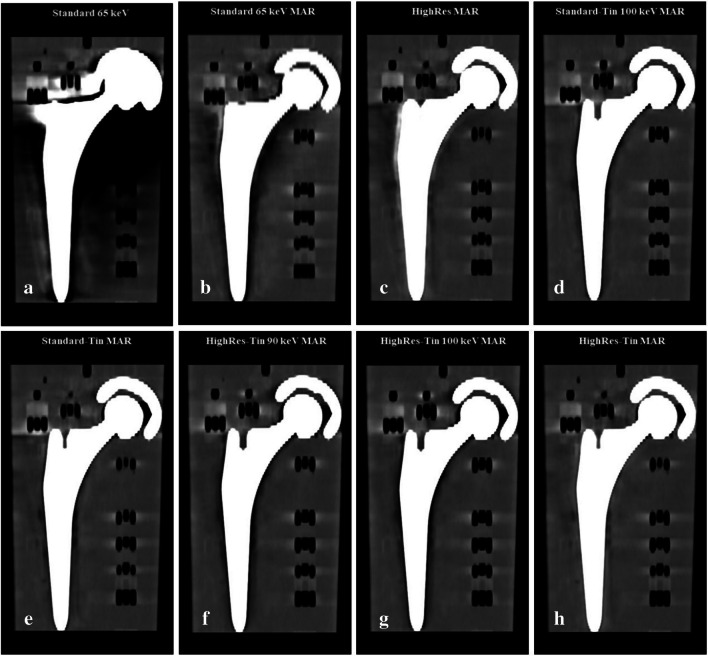
Coronal PCD CT images acquired with (**a**) Standard 65 keV (factory preset); **b** Standard 65 keV MAR; **c** HighRes MAR; **d** Standard-Tin 100 keV MAR, (**e**) Standard-Tin MAR; **f** HighRes-Tin 90 keV MAR; **g** HighRes-Tin 100 keV MAR; and (**h**) HighRes-Tin MAR. The selected images (**b-h**) with the parameters described above consistently achieved superior image quality compared with the reference image (**a**) and were rated equally as the best in terms of overall image quality. Standard = Quantumplus; Standard-Tin = QuantumSn; HighRes = UHR Q + ; HighRes-Tin = UHR QuantumSn; PCD CT = photon-counting detector computed tomography; Sn = tin filter; UHR = ultra-high resolution; VMI = virtual monoenergetic imaging

Only a minority of the images were preferred over the reference image (26.5%, 35/132). This included all polychromatic images with MAR as well as polychromatic Standard-Tin and HighRes-Tin. The results for the VMI images are more diverse. The monoenergy level range of the images preferred over the reference image ranged between 50 and 120 keV. No VMIs with or without MAR in the higher monoenergy spectra (130 – 190 keV) were preferred over the reference image. Tin-filter acquisitions (Standard-Tin) with and without MAR at the factory preset monoenergy level were preferred over the reference image. For the images without the tin filter (Standard) at the factory preset monoenergy level of 65 keV, only those processed with MAR were preferred over the reference image. Images acquired with the following modes were preferred over the reference image and scored "2" for artifacts, prosthesis delineation and synthetic image: VMI Standard 65 keV MAR, polychromatic HighRes MAR, VMI Standard-Tin 100 keV MAR, polychromatic Standard-Tin MAR, VMI HighRes-Tin 90 keV, HighRes-Tin 100 keV, as well as polychromatic HighRes-Tin (Fig. 5).

## Discussion

This study compared various modes for clinical PCD CT of a total hip prosthesis phantom, showing that the strongest artifact reduction was achieved by combining tin-filter, UHR and MAR for both for VMI and polychromatic images.

In both UHR and non-UHR acquisitions involving polychromatic image reconstructions, the use of a tin filter resulted in 14% decrease in the total artifact volume in our study. This effect is primarily a result of the combination of the tin filter, which eliminates low-energy photons, and the PCD CT detector technology, which eliminates electronic (background) noise. These observations are consistent with the phantom study of Anhaus et al. acquired on a PCD CT, where adding a tin filter resulted in only a minor reduction in metal artifacts for dental fillings, spine and hip implants [19]. However, a comparative study by Zhou et al. reported a significant 26% reduction in metal artifacts in a phantom study involving pedicle screws using a prototype PCD CT system [14]. Compared to clinical PCD CT, where typically either a monochromatic image is reconstructed at an energy level between 40 – 190 keV or a low-energy polychromatic image is reconstructed (photon energies of 20– 120 keV), Zhou et al. used high-energy threshold-bin reconstructions. Specifically, they utilized polychromatic reconstructions that encompass high-energy photons between 65 and 140 keV [14], which results in a relative lack of low-energy photons (20 – 64 keV), that are predominantly responsible for the metal artifacts. This results in an inherent reduction of metal artifacts, in addition to the spectral sharpening properties of the tin filter mentioned earlier.

For VMI reconstruction, the use of a tin filter led to an increase in the metal artifact volume at lower energies. The reason for the larger metal artifact volumes is the photon starvation effect, which becomes stronger at lower VMI energies when using the tin filter because of the impaired photon count. As a result of the energetic extrapolation in the VMI, this effect also carries over to energies above the peak energy of the spectrum, which corresponds to the observations in prior studies [27] and [19].

Images acquired in UHR mode showed slightly increased metal artifacts by 4% when compared to acquisitions without UHR. This is a novel observation, which is likely due to the UHR PCD design with an effective detector-element area of 0.25 mm × 0.25 mm (vs. 0.5 mm × 0.5 mm), which enables enhanced photon detection and generates a minimally larger artifact volume. However, when processed with MAR, the total artifact volume in images acquired in the UHR mode was smaller than in images acquired without the UHR mode. This is likely a result from the current MAR correction parameterization which were optimized for the non-UHR resolution.

In addition, we observed that MAR significantly reduced hypo-attenuated artifacts, but simultaneously increased the hyper-attenuated artifact volume. Two effects contribute to this phenomenon: First, the HU-based thresholding used to calculate the prior image within MAR can misclassify hyper-attenuated artifacts as bone-like areas, thereby enhancing them in the MAR correction loop. This misclassification is most likely to occur in regions near the metallic implant. Second, non-optimal sinogram normalization due to incomplete tissue classification can intensify hyper-attenuated artifacts across different rotation angles, as previously described [28]. These issues can currently not be mitigated without advanced prior image calculation techniques. Hypo-attenuating artifacts are mostly unaffected by this behavior, because air is more easily detected with the prior image, reducing the likelihood of misclassification.

In a retrospective PCD CT study of 33 patients with hip prostheses, Layer et al. [17] reported that the combination of VMI at 100 keV and iMAR was superior to polychromatic imaging with iMAR. Similar observations regarding dental implants have been made by Patzer et al. [16], who found an improved performance of VMI at 110 keV with iMAR in comparison to polychromatic reconstruction with iMAR. Additionally, Rau et al. [13] suggested VMI acquisition at 130 keV for assessing the spine with metal implants.

From a subjective image quality perspective, these observations are in line with our findings. However, from a quantitative perspective, our findings indicate that the presented optimal monoenergy levels are slightly higher. A potential explanation could be the use of a lower tube potential of 120 kV compared to 140 kV in our study. A higher tube potential consistently resulted in a reduced artifact volume. Additionally, we differentiated hyper- and hypo-attenuated artifact volumes, which provided more objective information about artifact geometry and propagation, when compared to the conventional quantification of corrected hyper- and hypo-attenuated artifacts.

Although MAR significantly and consistently reduced artifact volumes, it added new artifacts, such as synthetic image impressions. This is primarily due to overcorrection, which has already been described in PCD and EID CT studies [16, 17, 29]. Furthermore, metal artifact reduction software tends to generate areas of pseudo-osteolysis, that is, false osteolytic lesions at the bone-metal interface [27]. Although most of the images preferred over the reference image were reconstructed with MAR, evaluating non-MAR-generated images is therefore equally important.

Our study has limitations. We did not test different prosthesis sizes, nor did we vary the overall phantom size, as the primary objective was to assess the impact of various acquisition and reconstruction parameters on artifact volume and image quality. Furthermore, we refrained from embedding the prosthesis in bone material, as our aim was to evaluate the artifact volumes in their purest form. In addition, we evaluated 2 mm image slices, and we acknowledge that thicker slices inherently reduce metal artifacts through mechanisms such as the partial volume effect and beam hardening mitigation. Lastly, we did not evaluate the effect of the bone window on the artifacts, since at this point, MAR is only compatible with soft-bone kernels up to a maximum of B56.

In summary, VMI or polychromatic images obtained using a tin filter and UHR mode in combination with MAR led to the smallest artifact volumes and best image quality.

## Supplementary Information

Below is the link to the electronic supplementary material.

Supplementary file1 (DOCX 588 KB)

## Data Availability

Data is available on request.
