# Supplementary material for: Reducing Metal Artifacts in Clinical Photon Counting Detector Computed Tomography—A Phantom Study of an Exemplary Total Hip Arthroplasty
Source: Skeletal Radiol. 2024 Nov 19;54(6):1233–46. doi: 10.1007/s00256-024-04820-2 (PMC12000155; doi:10.1007/s00256-024-04820-2)
Supplement: Supplementary file 1 — Supplementary file1 (DOCX 588 KB) [file 256_2024_4820_MOESM1_ESM.docx]

**Supplementary Figures**


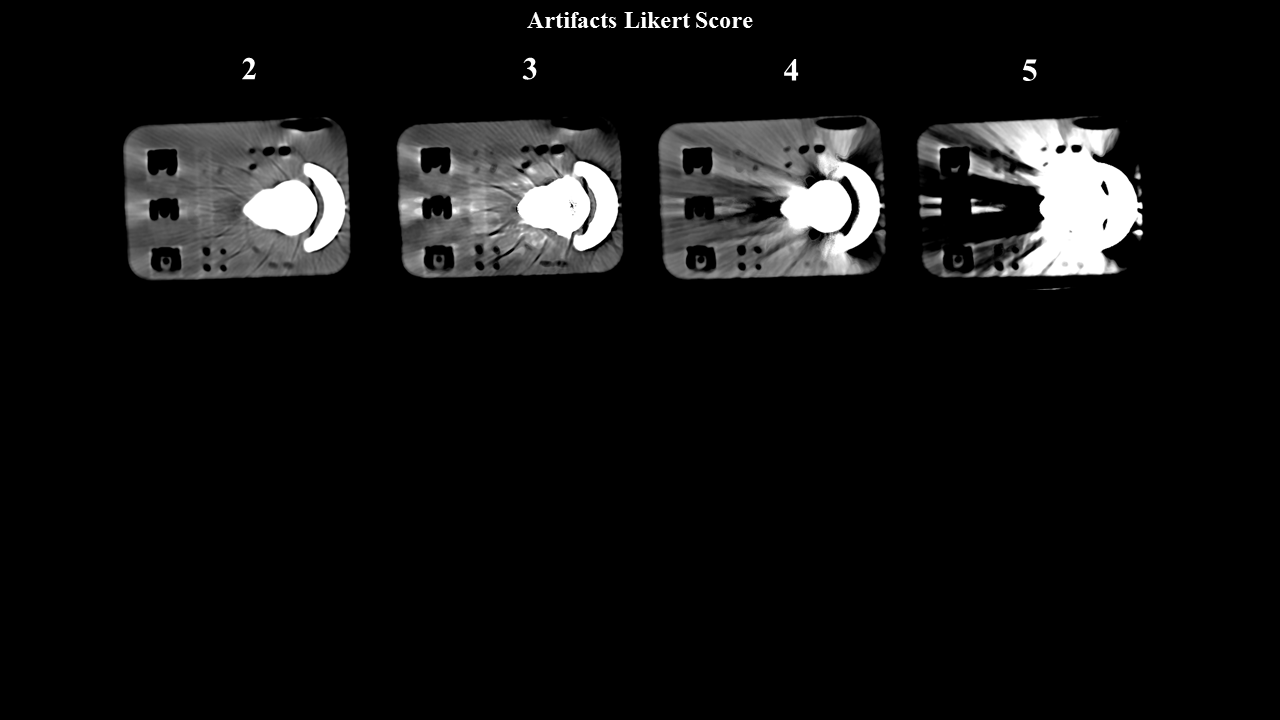


**Supplementary Figure 1.** Exemplary Likert score image series showing streak artifacts in axial PCD CT images cutting through the neck of the total hip prosthesis phantom. None of the acquisitions showed no artifact (Likert score 1). Likert score 2 = Minimal streak artifacts with no effect on surrounding structures. Likert score 3 = Minimal streak artifacts that affect the image quality. Likert score 4 = Moderate streak artifacts that considerably affect the image quality. Likert Score 5 = Severe streak artifacts that significantly affect the image quality. PCD CT, photon-counting detector computed tomography.


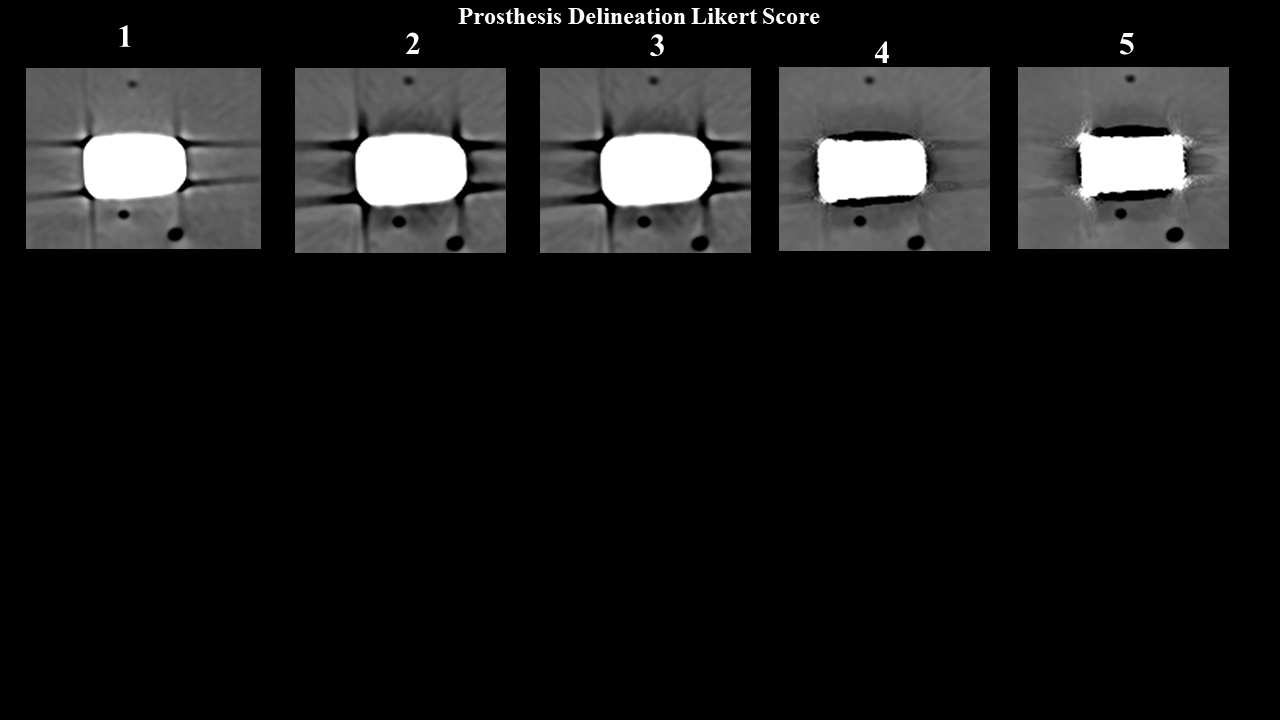


**Supplementary Figure 2.** Exemplary Likert score PCD CT image series of the hip prosthesis delineation in axial images cutting through the shaft of the prosthesis. Likert score 1 = Prosthesis is perfectly delineated. Likert score 2 = Minimal impairment that does not affect prosthesis delineation. Likert score 3 = Minimal impairment that affects prosthesis delineation. Likert score 4 = Moderate impairment that considerably affects prosthesis delineation. Likert score 5 = Severe impairment that significantly affects prosthesis delineation. Note that the magnification factor is increased threefold compared to Supplementary Figure 1. PCD CT, photon-counting detector computed tomography.


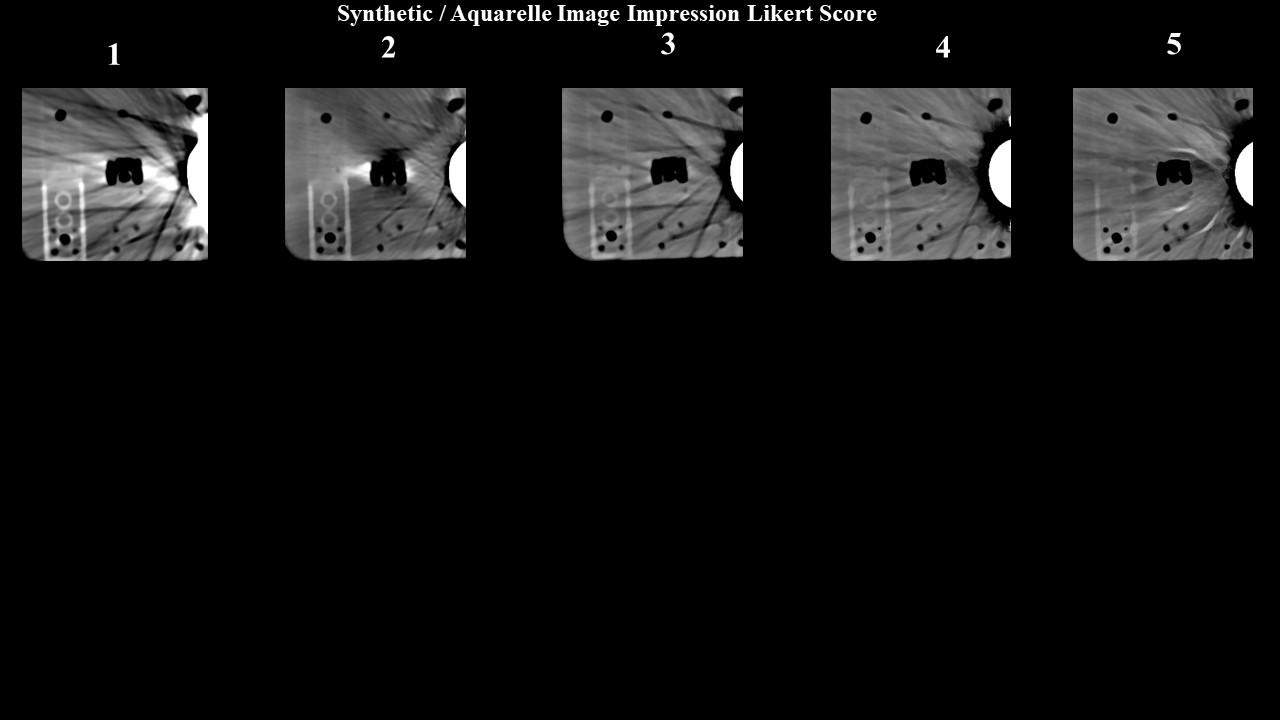
**Supplementary Figure 3.** Exemplary Likert score PCD CT image series of synthetic / aquarelle image impression in axial images that were acquired at the level of the prosthesis neck. Likert score 1 = no synthetic / aquarelle-like image impression. Likert score 2 = minimal synthetic / aquarelle-like image impression which does not affect the image quality. Likert score 3 = minimal synthetic / aquarelle-like image impression which does affect the image quality. Likert score 4 = moderate synthetic / aquarelle-like image impression which does considerably affect the image quality. Likert score 5 = severe synthetic / aquarelle-like image impression which does significantly affect the image quality. Note that the magnification factor is increased threefold compared to Supplementary Figure 1. PCD CT, photon-counting detector computed tomography.

**Supplementary Tables**

| **keV** | **Standard** | **Standard MAR** | **HighRes** | **HighRes MAR** | **Standard-Tin** | **Standard-Tin** | **HighRes-Tin** | **HighRes-Tin MAR** |
| --- | --- | --- | --- | --- | --- | --- | --- | --- |
| **Polychromatic** | X | X | X | X | X | X | X | X |
| **40** | X | X | X | X |  |  |  |  |
| **50** | X | X | X | X |  |  |  |  |
| **60** | X | X | X | X | X | X | X | X |
| **65** | X | X |  |  |  |  |  |  |
| **70** | X | X | X | X | X | X | X | X |
| **80** | X | X | X | X | X | X | X | X |
| **85** |  |  |  |  | X | X |  |  |
| **90** | X | X | X | X | X | X | X | X |
| **100** | X | X | X | X | X | X | X | X |
| **110** | X | X | X | X | X | X | X | X |
| **120** | X | X | X | X | X | X | X | X |
| **130** | X | X | X | X | X | X | X | X |
| **140** | X | X | X | X | X | X | X | X |
| **150** | X | X | X | X | X | X | X | X |
| **160** | X | X | X | X | X | X | X | X |
| **170** | X | X | X | X | X | X | X | X |
| **180** | X | X | X | X | X | X | X | X |
| **190** | X | X | X | X | X | X | X | X |

**Supplementary Table 1.** A total of 132 images were assessed. Polychromatic reconstruction was performed for all acquisitions. VMI acquired with tin filter (Sn) covered a monoenery range of 60 – 190 keV, while VMI acquired without tin filter encompassed the entire spectrum of 40 – 190 keV. In addition, VMI of Q+ and Q-SnSn were reconstructed at their respective factory preset optimal monoenergy levels of 65 keV and 85 keV, respectively. Standard = Quantumplus; Standard-Tin = QuantumSn; HighRes = UHR Q+; HighRes-Tin = UHR QuantumSn; Sn = tin filter; VMI = Virtual monoenergetic images; UHR = ulta-high resolution; MAR = iterative metal artifact reduction.

| Protocol | keV | Artifacts | | | | | | | | Prosthesis delineation | | | | | | | | Synthetic image impression | | | | | | | |
| --- | --- | --- | --- | --- | --- | --- | --- | --- | --- | --- | --- | --- | --- | --- | --- | --- | --- | --- | --- | --- | --- | --- | --- | --- | --- |
| Standard |  | Without MAR | | | | With MAR | | | | Without MAR | | | | With MAR | | | | Without MAR | | | | With MAR | | | |
|  |  | **R1** | **R2** | **R3** | **R4** | **R1** | **R2** | **R3** | **R4** | **R1** | **R2** | **R3** | **R4** | **R1** | **R2** | **R3** | **R4** | **R1** | **R2** | **R3** | **R4** | **R1** | **R2** | **R3** | **R4** |
|  | 40 | 4 | 4 | 4 | 4 | 3 | 4 | 4 | 4 | 5 | 5 | 5 | 5 | 4 | 5 | 4 | 4 | 5 | 5 | 5 | 5 | 5 | 5 | 5 | 5 |
|  | 50 | 5 | 4 | 4 | 5 | 3 | 3 | 4 | 4 | 5 | 5 | 5 | 5 | 4 | 4 | 4 | 4 | 2 | 2 | 3 | 4 | 3 | 3 | 3 | 4 |
|  | 60 | 5 | 5 | 5 | 5 | 3 | 3 | 4 | 3 | 5 | 5 | 5 | 5 | 3 | 3 | 3 | 3 | 2 | 2 | 2 | 2 | 3 | 3 | 3 | 3 |
|  | 65 | 4 | 5 | 4 | 4 | 2 | 2 | 2 | 2 | 4 | 4 | 5 | 5 | 2 | 2 | 2 | 2 | 1 | 1 | 1 | 1 | 2 | 2 | 1 | 1 |
|  | 70 | 4 | 4 | 4 | 4 | 3 | 3 | 3 | 3 | 5 | 5 | 5 | 5 | 3 | 3 | 3 | 3 | 2 | 2 | 2 | 2 | 2 | 2 | 2 | 2 |
|  | 80 | 4 | 4 | 4 | 4 | 3 | 3 | 3 | 3 | 4 | 5 | 5 | 5 | 3 | 3 | 3 | 3 | 2 | 2 | 2 | 2 | 2 | 2 | 2 | 2 |
|  | 90 | 4 | 4 | 4 | 4 | 3 | 3 | 3 | 3 | 4 | 4 | 5 | 4 | 3 | 3 | 4 | 4 | 3 | 2 | 2 | 2 | 2 | 2 | 2 | 2 |
|  | 100 | 4 | 4 | 5 | 4 | 3 | 3 | 4 | 3 | 5 | 4 | 5 | 5 | 4 | 4 | 4 | 4 | 4 | 4 | 4 | 4 | 3 | 3 | 3 | 4 |
|  | 110 | 5 | 5 | 5 | 4 | 3 | 3 | 3 | 3 | 5 | 5 | 5 | 5 | 4 | 4 | 4 | 4 | 4 | 4 | 4 | 4 | 4 | 4 | 4 | 4 |
|  | 120 | 5 | 5 | 5 | 4 | 3 | 3 | 3 | 3 | 5 | 5 | 5 | 5 | 4 | 4 | 4 | 4 | 5 | 5 | 5 | 5 | 4 | 4 | 4 | 4 |
|  | 130 | 5 | 5 | 5 | 5 | 3 | 3 | 3 | 3 | 5 | 5 | 5 | 5 | 4 | 4 | 4 | 4 | 5 | 5 | 5 | 5 | 4 | 4 | 4 | 4 |
|  | 140 | 5 | 5 | 5 | 5 | 3 | 3 | 4 | 3 | 5 | 5 | 5 | 5 | 4 | 4 | 4 | 4 | 5 | 5 | 5 | 5 | 4 | 4 | 4 | 4 |
|  | 150 | 5 | 5 | 5 | 5 | 3 | 3 | 4 | 3 | 5 | 5 | 5 | 5 | 4 | 4 | 4 | 4 | 5 | 5 | 5 | 5 | 4 | 4 | 4 | 4 |
|  | 160 | 5 | 5 | 5 | 5 | 3 | 3 | 3 | 3 | 5 | 5 | 5 | 5 | 4 | 5 | 5 | 4 | 5 | 5 | 5 | 5 | 4 | 4 | 4 | 4 |
|  | 170 | 5 | 5 | 5 | 5 | 3 | 3 | 3 | 4 | 5 | 5 | 5 | 5 | 4 | 5 | 5 | 5 | 5 | 5 | 5 | 5 | 4 | 5 | 5 | 5 |
|  | 180 | 5 | 5 | 5 | 5 | 3 | 3 | 4 | 4 | 5 | 5 | 5 | 5 | 5 | 5 | 5 | 5 | 5 | 5 | 5 | 5 | 5 | 5 | 5 | 5 |
|  | 190 | 5 | 5 | 5 | 5 | 3 | 3 | 4 | 4 | 5 | 5 | 5 | 5 | 5 | 5 | 5 | 5 | 5 | 5 | 5 | 5 | 5 | 5 | 5 | 5 |
|  | Polychromatic | 4 | 4 | 4 | 5 | 2 | 2 | 2 | 2 | 4 | 4 | 4 | 4 | 4 | 4 | 4 | 4 | 1 | 1 | 1 | 1 | 2 | 2 | 2 | 2 |

**Supplementary Table 2.**  Per reader visual assessment of artifacts, prosthesis delineation, and synthetic image impression in Standard acquisitions with and without MAR. Evaluation was performed using a 5-point Likert score, where a score of 1 denotes *no streak artifacts* in artifacts, *perfectly delineated prosthesis* in the prosthesis delineation category, and *no synthetic/aquarelle-like image impression* in the synthetic image impression category. A score of 5 signified the presence of *severe streak artifacts that significantly affected the image quality* in the artifact category, *severe impairment that significantly affected the prosthesis delineation* in the prosthesis delineation category, and *severe synthetic/aquarelle-like image impression, which significantly affected the image quality* in synthetic image quality. R1 = Fellowship trained radiologist with 10 years of clinical experience, R2 = Fellowship trained radiologist with 6 years of clinical experience, R3 = Subspecialty fellow with 5 years of clinical experience and R4 = Subspecialty fellow with 5 years of clinical experience. Standard = Quantumplus; MAR = iterative metal artifact reduction.

| Protocol | keV | Artifacts | | | | | | | | Prosthesis delineation | | | | | | | | Synthetic image impression | | | | | | | |
| --- | --- | --- | --- | --- | --- | --- | --- | --- | --- | --- | --- | --- | --- | --- | --- | --- | --- | --- | --- | --- | --- | --- | --- | --- | --- |
| HighRes |  | Without MAR | | | | With MAR | | | | Without MAR | | | | With MAR | | | | Without MAR | | | | With MAR | | | |
|  |  | **R1** | **R2** | **R3** | **R4** | **R1** | **R2** | **R3** | **R4** | **R1** | **R2** | **R3** | **R4** | **R1** | **R2** | **R3** | **R4** | **R1** | **R2** | **R3** | **R4** | **R1** | **R2** | **R3** | **R4** |
|  | 40 | 5 | 5 | 5 | 5 | 3 | 3 | 3 | 4 | 5 | 5 | 5 | 5 | 3 | 4 | 4 | 4 | 5 | 5 | 5 | 5 | 4 | 4 | 4 | 4 |
|  | 50 | 5 | 5 | 5 | 5 | 3 | 3 | 3 | 4 | 5 | 5 | 5 | 5 | 4 | 4 | 4 | 4 | 5 | 5 | 5 | 5 | 4 | 4 | 4 | 4 |
|  | 60 | 4 | 5 | 5 | 5 | 3 | 3 | 3 | 3 | 4 | 5 | 5 | 4 | 4 | 4 | 4 | 4 | 3 | 3 | 3 | 3 | 4 | 4 | 4 | 4 |
|  | 70 | 4 | 4 | 4 | 5 | 3 | 3 | 3 | 3 | 4 | 5 | 4 | 4 | 3 | 3 | 4 | 3 | 2 | 2 | 2 | 2 | 3 | 3 | 4 | 4 |
|  | 80 | 4 | 4 | 4 | 4 | 2 | 2 | 3 | 2 | 4 | 4 | 4 | 4 | 2 | 2 | 2 | 2 | 2 | 2 | 2 | 2 | 3 | 3 | 3 | 3 |
|  | 90 | 4 | 4 | 4 | 4 | 2 | 2 | 2 | 2 | 4 | 4 | 4 | 4 | 2 | 2 | 2 | 2 | 3 | 3 | 3 | 4 | 3 | 3 | 3 | 3 |
|  | 100 | 5 | 5 | 4 | 5 | 3 | 3 | 3 | 3 | 4 | 4 | 4 | 4 | 3 | 4 | 3 | 4 | 4 | 4 | 4 | 4 | 3 | 3 | 3 | 3 |
|  | 110 | 5 | 5 | 4 | 4 | 3 | 3 | 3 | 3 | 4 | 4 | 5 | 4 | 4 | 4 | 3 | 4 | 4 | 4 | 4 | 4 | 4 | 4 | 4 | 3 |
|  | 120 | 5 | 5 | 5 | 5 | 3 | 3 | 3 | 3 | 5 | 5 | 5 | 5 | 4 | 4 | 4 | 4 | 5 | 5 | 5 | 5 | 4 | 4 | 4 | 4 |
|  | 130 | 5 | 5 | 5 | 5 | 3 | 3 | 3 | 4 | 5 | 5 | 5 | 5 | 4 | 4 | 4 | 4 | 5 | 5 | 5 | 5 | 4 | 4 | 4 | 4 |
|  | 140 | 5 | 5 | 5 | 5 | 3 | 3 | 3 | 4 | 5 | 5 | 5 | 5 | 4 | 4 | 4 | 5 | 5 | 5 | 5 | 5 | 5 | 5 | 5 | 5 |
|  | 150 | 5 | 5 | 5 | 5 | 4 | 4 | 3 | 4 | 5 | 5 | 5 | 5 | 5 | 5 | 5 | 5 | 5 | 5 | 5 | 5 | 5 | 5 | 5 | 5 |
|  | 160 | 5 | 5 | 5 | 5 | 4 | 4 | 4 | 4 | 5 | 5 | 5 | 5 | 5 | 5 | 5 | 5 | 5 | 5 | 5 | 5 | 5 | 5 | 5 | 5 |
|  | 170 | 5 | 5 | 5 | 5 | 4 | 4 | 4 | 4 | 5 | 5 | 5 | 5 | 5 | 5 | 5 | 5 | 5 | 5 | 5 | 5 | 5 | 5 | 5 | 5 |
|  | 180 | 5 | 5 | 5 | 5 | 4 | 4 | 4 | 4 | 5 | 5 | 5 | 5 | 5 | 5 | 5 | 5 | 5 | 5 | 5 | 5 | 5 | 5 | 5 | 5 |
|  | 190 | 5 | 5 | 5 | 5 | 4 | 4 | 4 | 4 | 5 | 5 | 5 | 5 | 5 | 5 | 5 | 5 | 5 | 5 | 5 | 5 | 5 | 5 | 5 | 5 |
|  | Polychromatic | 4 | 4 | 4 | 5 | 2 | 2 | 2 | 2 | 4 | 4 | 4 | 4 | 2 | 2 | 2 | 2 | 1 | 1 | 2 | 2 | 2 | 1 | 2 | 2 |

**Supplementary Table 3.**  Per reader visual assessment of artifacts, prosthesis delineation, and synthetic image impression in HighRes acquisitions with and without MAR. Evaluation was performed using a 5-point Likert score, where a score of 1 denotes *no streak artifacts* in artifacts, *perfectly delineated prosthesis* in the prosthesis delineation category, and *no synthetic/aquarelle-like image impression* in the synthetic image impression category. A score of 5 signified the presence of *severe streak artifacts that significantly affected the image quality* in the artifact category, *severe impairment that significantly affected the prosthesis delineation* in the prosthesis delineation category, and *severe synthetic/aquarelle-like image impression that significantly affected the image quality* in synthetic image quality. R1 = Fellowship trained radiologist with 10 years of clinical experience, R2 = Fellowship trained radiologist with 6 years of clinical experience, R3 = Subspecialty fellow with 5 years of clinical experience and R4 = Subspecialty fellow with 5 years of clinical experience HighRes = UHR Quantumplus; UHR = ultra-high resolution; MAR = iterative metal artifact reduction.

| Protocol | keV | Artifacts | | | | | | | | Prosthesis delineation | | | | | | | | Synthetic image impression | | | | | | | |
| --- | --- | --- | --- | --- | --- | --- | --- | --- | --- | --- | --- | --- | --- | --- | --- | --- | --- | --- | --- | --- | --- | --- | --- | --- | --- |
| Standard-Tin |  | Without MAR | | | | With MAR | | | | Without MAR | | | | With MAR | | | | Without MAR | | | | With MAR | | | |
|  |  | **R1** | **R2** | **R3** | **R4** | **R1** | **R2** | **R3** | **R4** | **R1** | **R2** | **R3** | **R4** | **R1** | **R2** | **R3** | **R4** | **R1** | **R2** | **R3** | **R4** | **R1** | **R2** | **R3** | **R4** |
|  | 60 | 5 | 5 | 5 | 5 | 3 | 3 | 3 | 3 | 5 | 5 | 4 | 4 | 2 | 2 | 2 | 2 | 3 | 3 | 3 | 2 | 4 | 4 | 4 | 4 |
|  | 70 | 5 | 5 | 5 | 5 | 4 | 4 | 4 | 4 | 4 | 5 | 4 | 4 | 2 | 2 | 2 | 2 | 2 | 2 | 3 | 3 | 3 | 3 | 3 | 3 |
|  | 80 | 4 | 5 | 4 | 4 | 3 | 3 | 3 | 3 | 4 | 4 | 4 | 4 | 2 | 2 | 2 | 2 | 2 | 2 | 2 | 2 | 3 | 3 | 3 | 3 |
|  | 85 | 4 | 4 | 4 | 3 | 3 | 4 | 3 | 3 | 4 | 4 | 3 | 4 | 2 | 2 | 2 | 2 | 2 | 2 | 2 | 2 | 2 | 2 | 2 | 2 |
|  | 90 | 4 | 4 | 4 | 4 | 3 | 3 | 3 | 3 | 4 | 4 | 4 | 4 | 2 | 2 | 2 | 2 | 2 | 2 | 2 | 2 | 2 | 2 | 2 | 2 |
|  | 100 | 3 | 3 | 3 | 4 | 2 | 3 | 2 | 2 | 3 | 3 | 4 | 4 | 2 | 2 | 2 | 2 | 2 | 2 | 2 | 4 | 2 | 2 | 2 | 2 |
|  | 110 | 3 | 3 | 3 | 3 | 3 | 3 | 2 | 3 | 4 | 3 | 4 | 3 | 3 | 3 | 2 | 3 | 3 | 3 | 3 | 4 | 3 | 3 | 3 | 3 |
|  | 120 | 3 | 3 | 3 | 3 | 3 | 3 | 3 | 3 | 4 | 3 | 4 | 4 | 3 | 3 | 3 | 3 | 4 | 3 | 4 | 4 | 3 | 3 | 3 | 3 |
|  | 130 | 4 | 4 | 4 | 5 | 3 | 3 | 3 | 3 | 5 | 4 | 4 | 4 | 3 | 4 | 3 | 4 | 4 | 4 | 4 | 4 | 4 | 4 | 3 | 4 |
|  | 140 | 5 | 5 | 4 | 5 | 3 | 3 | 3 | 3 | 5 | 4 | 5 | 5 | 4 | 4 | 4 | 4 | 5 | 5 | 5 | 4 | 4 | 4 | 4 | 4 |
|  | 150 | 5 | 5 | 5 | 5 | 3 | 3 | 3 | 3 | 5 | 5 | 5 | 5 | 4 | 4 | 4 | 4 | 5 | 5 | 5 | 5 | 4 | 4 | 4 | 4 |
|  | 160 | 5 | 4 | 5 | 5 | 3 | 3 | 3 | 3 | 5 | 5 | 5 | 5 | 4 | 4 | 4 | 4 | 5 | 5 | 5 | 5 | 4 | 4 | 4 | 4 |
|  | 170 | 5 | 5 | 5 | 5 | 3 | 3 | 3 | 4 | 5 | 5 | 5 | 5 | 4 | 4 | 4 | 4 | 5 | 5 | 5 | 5 | 4 | 4 | 4 | 4 |
|  | 180 | 5 | 5 | 5 | 5 | 3 | 3 | 4 | 4 | 5 | 5 | 5 | 5 | 4 | 4 | 4 | 4 | 5 | 5 | 5 | 5 | 4 | 4 | 4 | 4 |
|  | 190 | 5 | 5 | 5 | 5 | 4 | 3 | 4 | 4 | 5 | 5 | 5 | 5 | 4 | 4 | 4 | 4 | 5 | 5 | 5 | 5 | 4 | 4 | 4 | 4 |
|  | Polychromatic | 4 | 4 | 5 | 4 | 2 | 2 | 2 | 2 | 4 | 5 | 4 | 4 | 2 | 2 | 2 | 2 | 1 | 1 | 1 | 1 | 2 | 2 | 2 | 1 |

**Supplementary Table 4.**  Per reader visual assessment of artifacts, prosthesis delineation, and synthetic image impression in Standard-Tin acquisitions with and without MAR. Evaluation was performed using a 5-point Likert score, where a score of 1 denotes *no streak artifacts* in artifacts, *perfectly delineated prosthesis* in the prosthesis delineation category, and *no synthetic/aquarelle-like image impression* in the synthetic image impression category. A score of 5 signified the presence of *severe streak artifacts that significantly affected the image quality* in the artifact category, *severe impairment that significantly affected the prosthesis delineation* in the prosthesis delineation category, and *severe synthetic/aquarelle-like image impression that significantly affected the image quality* in synthetic image quality. R1 = Fellowship trained radiologist with 10 years of clinical experience, R2 = Fellowship trained radiologist with 6 years of clinical experience, R3 = Subspecialty fellow with 5 years of clinical experience and R4 = Subspecialty fellow with 5 years of clinical experience. Standard-Tin = QuantumSn, Sn = tin-filter; MAR = iterative metal artifact reduction.

| Protocol | keV | Artifacts | | | | | | | | Prosthesis delineation | | | | | | | | Synthetic image impression | | | | | | | |
| --- | --- | --- | --- | --- | --- | --- | --- | --- | --- | --- | --- | --- | --- | --- | --- | --- | --- | --- | --- | --- | --- | --- | --- | --- | --- |
| HighRes-Tin |  | Without iMAR | | | | With iMAR | | | | Without iMAR | | | | With iMAR | | | | Without iMAR | | | | With iMAR | | | |
|  |  | **R1** | **R2** | **R3** | **R4** | **R1** | **R2** | **R3** | **R4** | **R1** | **R2** | **R3** | **R4** | **R1** | **R2** | **R3** | **R4** | **R1** | **R2** | **R3** | **R4** | **R1** | **R2** | **R3** | **R4** |
|  | 60 | 5 | 5 | 5 | 5 | 4 | 4 | 5 | 4 | 4 | 5 | 5 | 4 | 4 | 5 | 4 | 4 | 2 | 2 | 2 | 2 | 4 | 3 | 3 | 3 |
|  | 70 | 5 | 5 | 5 | 5 | 4 | 4 | 4 | 4 | 5 | 5 | 5 | 5 | 4 | 4 | 4 | 4 | 2 | 2 | 2 | 2 | 3 | 3 | 3 | 3 |
|  | 80 | 4 | 4 | 4 | 4 | 3 | 3 | 3 | 3 | 4 | 4 | 4 | 4 | 3 | 4 | 4 | 3 | 2 | 2 | 2 | 2 | 3 | 3 | 3 | 3 |
|  | 90 | 4 | 4 | 4 | 4 | 2 | 2 | 2 | 3 | 4 | 4 | 4 | 4 | 2 | 2 | 2 | 2 | 2 | 2 | 2 | 2 | 2 | 2 | 2 | 2 |
|  | 100 | 3 | 4 | 3 | 4 | 2 | 2 | 2 | 2 | 3 | 4 | 4 | 3 | 2 | 2 | 2 | 2 | 2 | 2 | 2 | 2 | 2 | 2 | 2 | 2 |
|  | 110 | 3 | 3 | 3 | 3 | 3 | 2 | 3 | 3 | 3 | 4 | 4 | 3 | 3 | 3 | 3 | 3 | 3 | 3 | 2 | 2 | 3 | 3 | 3 | 2 |
|  | 120 | 3 | 3 | 3 | 3 | 3 | 3 | 3 | 3 | 4 | 4 | 4 | 3 | 3 | 3 | 3 | 3 | 4 | 4 | 4 | 4 | 3 | 3 | 3 | 3 |
|  | 130 | 3 | 3 | 3 | 3 | 3 | 3 | 3 | 3 | 4 | 4 | 4 | 4 | 3 | 3 | 4 | 3 | 4 | 4 | 4 | 4 | 3 | 3 | 3 | 3 |
|  | 140 | 4 | 4 | 4 | 3 | 3 | 3 | 3 | 3 | 4 | 4 | 4 | 4 | 3 | 4 | 4 | 3 | 4 | 4 | 4 | 4 | 4 | 4 | 4 | 4 |
|  | 150 | 4 | 4 | 4 | 4 | 3 | 3 | 4 | 3 | 4 | 4 | 4 | 4 | 4 | 4 | 4 | 4 | 4 | 4 | 4 | 4 | 4 | 4 | 4 | 4 |
|  | 160 | 5 | 4 | 5 | 4 | 3 | 3 | 4 | 3 | 5 | 5 | 4 | 5 | 4 | 4 | 4 | 4 | 5 | 5 | 5 | 4 | 4 | 4 | 4 | 4 |
|  | 170 | 5 | 5 | 5 | 5 | 4 | 3 | 4 | 4 | 5 | 5 | 5 | 5 | 4 | 4 | 4 | 4 | 5 | 5 | 5 | 5 | 4 | 4 | 4 | 4 |
|  | 180 | 5 | 5 | 5 | 5 | 4 | 4 | 4 | 4 | 5 | 5 | 5 | 5 | 4 | 4 | 4 | 4 | 5 | 5 | 5 | 5 | 4 | 4 | 4 | 4 |
|  | 190 | 5 | 5 | 5 | 5 | 4 | 4 | 4 | 4 | 5 | 5 | 5 | 5 | 4 | 4 | 4 | 4 | 5 | 5 | 5 | 5 | 4 | 4 | 4 | 4 |
|  | Polychromatic | 4 | 4 | 4 | 5 | 2 | 2 | 2 | 2 | 4 | 4 | 4 | 4 | 2 | 2 | 2 | 2 | 1 | 1 | 1 | 1 | 2 | 2 | 2 | 2 |

**Supplementary Table 5.**  Per reader visual assessment of artifacts, prosthesis delineation, and synthetic image impression in HighRes-Tin acquisitions with and without MAR. Evaluation was performed using a 5-point Likert score, where a score of 1 denotes *no streak artifacts* in artifacts, *perfectly delineated prosthesis* in the prosthesis delineation category, and *no synthetic/aquarelle-like image impression* in the synthetic image impression category. A score of 5 signified the presence of *severe streak artifacts that significantly affected the image quality* in the artifact category, *severe impairment that significantly affected the prosthesis delineation* in the prosthesis delineation category, and *severe synthetic/aquarelle-like image impression that significantly affected the image quality* in synthetic image quality. R1 = Fellowship trained radiologist with 10 years of clinical experience, R2 = Fellowship trained radiologist with 6 years of clinical experience, R3 = Subspecialty fellow with 5 years of clinical experience and R4 = Subspecialty fellow with 5 years of clinical experience. HighRes-Tin = QuantumSn; Sn = tin-filter; UHR = ultra-high resolution; MAR = iterative metal artifact reduction.
